# Restaurant customers’ food leftover reduction intention derived from nature connection and biospheric values: A comparison between men and women

**DOI:** 10.3389/fpsyg.2022.976102

**Published:** 2023-01-11

**Authors:** Wansoo Kim, Chen Che, Chul Jeong

**Affiliations:** ^1^Department of Tourism Management, Gachon University, Seongnam, Republic of Korea; ^2^College of History and Tourism Culture, Inner Mongolia University, Hohhot, China; ^3^Division of Tourism Science, Hanyang University, Seoul, Republic of Korea

**Keywords:** restaurant customer, nature connection, biospheric values, environmental self-identity, sense of obligation to reduce food leftover, food leftover reduction intention, gender comparison

## Abstract

As the COVID-19 pandemic extends over a long period of time, the World Food Programme (WFP) estimated that food insecurity would take place in the near future. Previous studies focused on various kinds of interventions for food waste prevention. Surprisingly, however, research tackling consumer attitudes and behaviors as a way to reduce food waste is still rare. To fill this gap in the literature, this study examined the antecedent roles of restaurant customers’ nature connection and biospheric values in fostering their food leftover reduction intention through environmental self-identity and sense of obligation to reduce food leftover. In addition, the moderating effects of gender were tested on all the relationships in our conceptual model. A quantitative approach with an online survey for restaurant customers was adopted. Structural equation modeling was adopted to analyze the data. Through confirmatory factor analyses, the adequate reliability and validity of the measures were established. All the relationships between the constructs were found to be significant, supporting the hypotheses. In other words, the restaurant customers’ nature connection and biospheric values were found to eventually induce the customers’ food leftover reduction intention. In addition, in terms of the moderating effect, the male customers’ nature connection more strongly increased their biospheric values than the female customers’ case. The findings of this study revealed how restaurant customers’ food leftover reduction intention is formed through their feeling of oneness with nature and biospheric values. Given that consumer behavior has been recognized as a major driver of restaurant food waste, the findings of this study provide useful insights to restauranteurs and policymakers for the health of society and people in it. It was especially true for men in that their feeling of oneness with nature significantly influences their biospheric values more than women’s.

## 1. Introduction

Food waste detrimentally affects the environment, for example, by increasing carbon emissions (Rico et al., 2019) and global water usage (Gössling, 2015). Food waste also leads to socioeconomic concerns such as global food insecurity (Godfray et al., 2010) and excessive global food price inflation (Quested et al., 2013). As the COVID-19 pandemic becomes protracted, World Food Programme (WFP) estimated that 271.8 million people will suffer from severe food insecurity (World Food Programme, 2022). South Korea has one of the world’s highest rates of food waste, and the primary reason is the banchan food culture (Earth, 2021). A Korean meal table is typically composed of bap (rice), kuk (soup), and various banchan (side dishes) on one table (Kim et al., 2016). Banchan comprises of kimchi, vegetable, and high-protein dishes (Kwon et al., 2015). It means people get many side dishes. Nowadays, Radio-frequency identification (RFID) technology applies in various industries (Öztayşi et al., 2009). Wen et al. (2018) explored the Internet of Things (IoT) network technology (which used RFID tags) to improve the management of restaurant food waste in China. Hong et al. (2014) improved the RFID-based garbage collection system to reduce food waste. Since 2013, Korea has installed radio-frequency identification (RFID) machines to encourage people to reduce food waste. Even though RFID machines have reduced 47,000 tons of food waste in the last 6 years, people still waste too much food (Food and Agriculture Organization of the United Nations, 2021). Hence, The Korea Zero Waste Movement Network (KZWMN) suggested changing the dining habits, especially reducing the number of banchan (i.e., side dishes in Korean food; KZWMN, 2022).

On top of that, previous studies have demonstrated that food waste affects the profitability of food service businesses by wasting food materials, energy, water, labor, and more. Christ and Burritt (2017) were the first researchers to use the material flow cost accounting (MFCA) tool to examine food waste in the restaurant industry. Bux and Amicarelli (2022) applied the MFCA tool to determine the material, energy, environmental, and economic costs of food waste. Principato et al. (2021) designed Restaurant Food Waste Map (RFWM) to explain outdoor food waste. This series of studies show that reducing food waste is one of the biggest challenges for the food service industry’s sustainable growth. Thus, the policymakers and businesses have clear motives to reduce food waste. The remaining key player in reducing restaurant food waste is the customer. Irresponsible consumer behaviors have been criticized as one of the major causes of restaurant food waste (Sakaguchi et al., 2018). If they can be successfully induced to reduce food leftover, the fight to prevent excessive food waste will be much easier and more effective.

The COVID-19 pandemic has allowed calm reflection on consumers’ dining behaviors (Amicarelli et al., 2021). In their study on Italian food consumers after the lockdown, Amicarelli et al. (2021) found that stay-at-home customers are likelier to perceive food waste and change their wasteful habits. Raising individuals’ awareness, attitudes, and dining behaviors at the household level is crucial in reducing food waste and improving the environmental, economic, and social impact (Secondi et al., 2015). Using the extended TPB model, Graham-Rowe et al. (2015) found that the household participants had the intention to reduce their fruit and vegetable waste. Previous research efforts have been devoted more to food waste from households than that from foodservices (Wang et al., 2017; Abdelradi, 2018; Adelodun et al., 2021; Ananda et al., 2021). The literature indicates that more food waste is generated at eating-out locations than at home (Amicarelli et al., 2021). The United Nations’ Sustainable Development Goals (SDGs) recently set a goal for halving global food waste by 2030 (Food and Agriculture Organization of the United Nations, 2022). Given that the frequency of eating out is continuously increasing globally (Evans, 2012; Thyberg and Tonjes, 2016; Schanes et al., 2018; Filimonau et al., 2019), the urgent need to change consumer behaviors in food services strongly emerges.

Researchers have recently begun to investigate what interventions for food waste prevention would be effective and what drives consumer food waste (Stöckli et al., 2018). Although some food management research has been carried out on consumers’ food waste reduction intention, there have been few empirical investigations into Korean consumers (Moraes et al., 2021). Accordingly, this study attempts to fill this gap in the literature by adopting restaurant customers’ nature connection, biospheric value, and environmental self-identity as the predictors of their sense of obligation to reduce food leftover and, in turn, their food leftover reduction intention. Previous research suggests that biospheric values can promote people’s tendency toward environment-friendly attitudes and intentions (Steg and de Groot, 2012). Oh et al. (2021) used a national survey in Singapore to establish that biospheric values had a direct and positive relationship with nature-relatedness. In this sense, this study’s objective was to reveal the promotive effects of nature connection and biospheric values in stimulating restaurant customers’ leftover reduction intention through environmental self-identity and a sense of obligation to reduce food leftover.

## 2. Literature review and hypotheses development

### 2.1. Food waste from customer plate

In the food service sector, food is often considered the least costly resource and is mainly regarded as disposable by restaurants (Garrone et al., 2014). Customer behavior has been recognized as a key driver of food waste in the food service context (Martin-Rios et al., 2018). Buzby et al. (2014) defined food waste as *“an edible item is discarded, such as plate waste discarded by consumers.”* They also said plate scraps that customers do not take home are disposed of properly in restaurants for health reasons (Buzby et al., 2014). Sakaguchi et al. (2018) reported that consumers’ attitudes and behaviors toward food waste played essential roles in deciding the amount of food discarded in restaurants. In the EU 28 countries, for example, the food service sector generates the third largest amount of food waste, right after households and farming/food processing industries (Katsarova, 2014), and around 75% of this food waste is considered avoidable (Oliveira et al., 2016). Several studies have indicated that education and policy positively impact food waste reduction behavior (Graham-Rowe et al., 2015; Pinto et al., 2018; Giordano et al., 2019). Several studies have examined waste minimization (Barr et al., 2001; Tonglet et al., 2004; Ertz et al., 2021). Barr et al. (2001) found that environmental values play an important part in waste minimization and reuse. Tonglet et al. (2004) discussed the householder attitude to minimize household waste. Ertz et al. (2021) applied the theory of planned behavior to analyze the consumer behavior of waste minimization. Graham-Rowe et al. (2014) used a qualitative research approach to identify the core motivation for food waste minimization. They pointed out that most households regarded food waste as financial waste. Some researchers have attempted to explain the relationships between demographic variables and waste minimization intention (Elhoushy, 2020). Wang et al. (2017) reviewed the clean plate campaigns such as “Clean Plate Club” in the US, “Clean Plate Movement” in Korea, and “China’s Clean Your Plate Campaign I, II” in China. Their result showed that cultural value is one of the driving factors of food waste. The most representative example is customers’ hospitality and face-saving behavior (Wang et al., 2017). It means that people in some culture waste food to show their hospitality to their guests. Much of the literature on food waste deals with the question of consumers’ food waste management (Von Kameke and Fischer, 2018; Attiq et al., 2021; Chen et al., 2021). Therefore, paradoxically it also implies that consumer behaviors can be changed by changing their traditional cultural belief through campaigns, education, policy, and so on. With a few changes in consumer behavior, food waste from the food service sector can be reduced considerably and its impact on environmental protection and food security will be substantial (Oliveira et al., 2016). Ordering just enough food to finish and taking leftover food home are good examples of simple but powerful consumer behaviors (Coşkun and Özbük, 2020). Therefore, it is evident that changing consumer behavior is a critical, effective, and efficient way to mitigate various negative environmental and socioeconomic impacts of food waste. In this regard, this study suggests customers’ nature connection and biospheric values as key determinants of their food leftover reduction intention and thus as the starting points to develop effective nudging interventions to prevent food leftover from customer plates.

### 2.2. Nature connection and biospheric values

Humans are assumed to have developed an innate affinity for nature through long evolutionary history in nature. Humans are in nature, and nature is in humans (Wilson, 1984). Humans love the wilderness, marine activities, zoos, aquariums, and natural scenery (Nisbet et al., 2009). Nature connection refers to one’s feeling of “oneness” with nature that stems from accepting nature as an important part of one’s self-definition (Mayer and Frantz, 2004). It is a relationship between a human and nature, which ecopsychology is largely focused on. Research has revealed that individuals who feel connected with nature tend to have higher levels of well-being such as life satisfaction (Capaldi et al., 2014), meaning-in-life (Howell et al., 2013), mindfulness (Schutte and Malouff, 2018), and more. Martin and Czellar (2017) were the first to discuss the relationship between nature connection and biospheric values. They analyzed cross-sectional data from the United States and Europe people. The result of their study showed that nature connection positively impacts biospheric values. In short, feeling connected with nature makes humans feel comfortable and happy so people have a fundamental need for a nature connection (Moreton et al., 2019). In this sense, from an evolutionary perspective, protecting nature also means protecting humans.

*Values* are beliefs that guide one’s decisions across various contexts and domains (Schwartz, 1977). Biospheric values refer to a value orientation where “people judge phenomena based on costs or benefits to ecosystems or the biosphere” (Stern and Dietz, 1994). Thus, those who have biospheric values tend to judge their own and others’ behaviors from the viewpoint of costs or benefits to nature (Martin and Czellar, 2017). Owing to this environmental relevance of biospheric values, biospheric values have been found to positively affect pro-environmental attitudes and behaviors (Steg et al., 2014). Therefore, it is critical to understand how biospheric values can be cultivated in citizens to promote their environment-friendly attitudes and behaviors (Martin and Czellar, 2017).

As mentioned, nature connection is defined as one’s feeling of oneness with nature. Thus, for those with a robust nature connection, it is natural to perceive the behavior harmful to nature as harmful to themselves. Accordingly, they are more likely to judge phenomena based on costs or benefits to nature, which is a characteristic of biospheric values. Thus, a hypothesis follows as below.

*H*1: Nature connection positively affects biospheric values.

### 2.3. Environmental self-identity

Self-identity refers to the way people label themselves. It is formed not only by one’s values and beliefs but also by others’ expectations or labels received through social interactions (Whitmarsh and O'Neill, 2010). Once accepted, self-identity guides one’s behaviors because people know that behavior is the most direct clue that shows who we are (i.e., our self-identity; Lacasse, 2016). In fact, self-identity has been verified to be a significant determinant of behavior over and above the variables in the Theory of Planned Behavior (TPB; Whitmarsh and O'Neill, 2010). The TPB argues that behavioral intention is formed by attitude toward a certain behavior, subjective norm, and perceived behavioral control (Ajzen, 1991). How we label ourselves more powerfully determines how we behave than the TPB variables do (Fekadu and Kraft, 2001). In terms of environmental labels, labeling themselves “environment-friendly” or “environmentalists” means accepting environmental self-identity, which is defined as the degree to which someone views oneself as a person who behaves pro-environmentally (Van der Werff et al., 2013a). Given that nature connection indicates the feeling of oneness with nature, those with a vital nature connection would care for the environment as they care for themselves. Thus, they should label themselves as “environment-friendly.” Therefore, the following hypothesis can be suggested.

*H*2: Nature connection positively affects environmental self-identity.

Values reflect what is considered vital in one’s life and thus affect how people see themselves and what kind of person they want to be (Van der Werff et al., 2013b). As such, values reflect self-concept or self-identity. As mentioned, biospheric values reflect the extent to which people judge behaviors based on costs or benefits to nature. Therefore, for those who have biospheric values, the behaviors that cause costs to nature are considered as undesirable, and thus they will refrain from such behaviors. On the other hand, the behaviors that are beneficial to nature are considered desirable, and thus they will actively engage in such behaviors. Those are the behavioral characteristics of people with environmental self-identity who label themselves as environment-friendly (Jans, 2021). Therefore, a hypothesis can be set as follows.

*H*3: Biospheric values positively affects environmental self-identity.

### 2.4. Sense of obligation to reduce food leftover and food leftover reduction intention

Environmental self-identity has been revealed to lead to pro-environmental attitudes and behaviors such as switching to green energy, purchasing environment-friendly goods, and using energy-saving transportation (Lacasse, 2016). Stancu and Lähteenmäki (2022) mentioned that consumers’ awareness of saving and environmental self-identity highly influences their motivation to reduce food waste. In the food service context, customers’ environmental self-identity would lead to their sense of obligation to reduce food leftover and, in turn, to food leftover reduction intention. The logic for self-identity’s effect on behavioral intention derives from identity theory (Stryker, 1980). The more apparent an identity is, the greater the likelihood of role-consistent behavior because not engaging in role-appropriate action may create internal tension due to conflict between identity and behavior (Fielding et al., 2008). For instance, people who consider themselves water savers are more likely to save water than others (Mannetti et al., 2004). As such, to avoid uncomfortable internal tension, people with salient environmental self-identity would feel more obligation to behave environment-friendly, reducing food leftover in the food service context. In turn, this sense of obligation to reduce food leftover, as a type of moral norm, would lead to actual food leftover reduction intention as norm leads to intention (Schutte and Malouff, 2018).

*H*4: Environmental self-identity positively affects sense of obligation to reduce food leftover.

*H*5: Sense of obligation to reduce food leftover positively affects food leftover reduction intention.

### 2.5. Gender differences

The socialization theory suggests that depending on gender, individuals experience different socialization processes (Baldwin, 2002). Their peer groups shape individuals’ socialization process. Parents, and other adults, have different expectations of them depending on gender (Kamau, 2020). Consequently, gender role theory proposes that males and females are grown with different expectations to carry out different gender roles in society (Shimanoff, 2009). From an evolutionary psychology perspective, men have been expected to go out and bring something to feed the family, which is money in modern times. In contrast, women have been expected to share food in the family or community and take care of children. As a result, men are evolved to be aggressive and competitive, while women are caring and cooperative in general (Birindelli et al., 2019). It appears that such different characteristics by gender are reflected in their responses to environmental issues.

**Figure 1:**
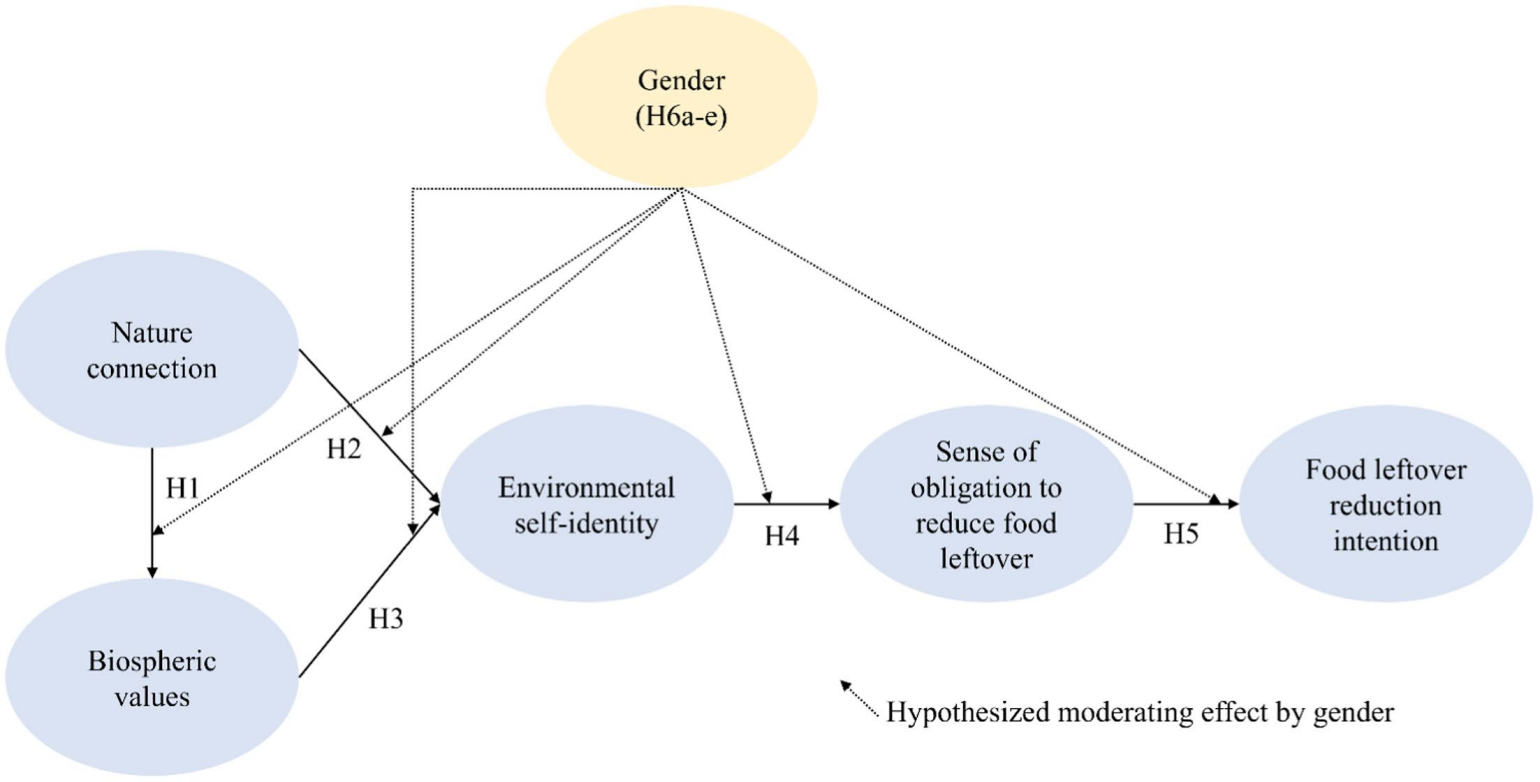
The proposed conceptual framework.

Some sociologists propose that women are more concerned about protecting the environment due to their reproductive role (Rand et al., 2016). To take care of babies from pregnancy to toddler, women should stay in a stable environment where no rapid, unexpected changes happen. In this sense, preserving the environment would be a more critical issue for women than men. The prior studies showed that females prefer to minimize their waste more than males (Barr et al., 2001; Graham-Rowe et al., 2015). However, no general agreement has yet been reached regarding gender differences in pro-environmental attitudes and behaviors in the literature (Briscoe et al., 2019). The current study considers moderating effects of gender on the hypothesized relationships in forming food leftover reduction intention because, as social entities, people’s attitudes and behaviors are influenced by different gender role expectations (Trelohan, 2022). In other words, this research will explore the moderating role of gender and explain the different attitudes and behavior between gender (Figure 1).

*H*6a–e: Gender plays a moderating role in the process of forming food leftover reduction intention.

## 3. Methodology

### 3.1. Measurement items and questionnaire

Questionnaire survey is one of the well-established approaches in food waste research, for example, household food waste questionnaires, food waste value questionnaires, and restaurant food waste questionnaires (Wang et al., 2017; Giordano et al., 2018, 2019; Amicarelli and Bux, 2020). Confirmatory factor analysis (CFA) is one testing prespecified measurement theory composed of measured items and factors that fit reality as represented by data. CFA is a precondition to conduct structural equation modeling (SEM; Thompson, 2004; Hair et al., 2010), which is to test a series of relationships between independent variables and dependent variables to verify hypotheses (Thompson, 2004; Ullman and Bentler, 2012; Hair et al., 2014). For this study, well-validated measurement items were borrowed from preceding research to evaluate the constructs. In detail, eight measurement items were employed for nature connection (e.g., “I often feel a sense of oneness with the natural world around me”; Mayer and Frantz, 2004). In order to measure biospheric values, four items were employed (e.g., “We human beings should live harmoniously with the nature” (Stern et al., 1999). Environmental self-identity was assessed with three items (e.g., “Acting environmentally-friendly is an important part of who I am”; Van der Werff et al., 2013a). Four items were utilized to measure sense of obligation to reduce food waste (e.g., “I feel guilty about poor people when I leave leftover food”; Visschers et al., 2016). Lastly, in order to measure food leftover reduction intention, three items were used (e.g., “I intend to not throw food away”; Visschers et al., 2016). All the measures were evaluated with a 7-point Likert scale ranging from “Strongly disagree” (1) to “Strongly agree” (7). In addition to the measures introduced so far, the questionnaire consisted of a research description and questions asking survey participants’ demographic information. A pre-test for the questionnaire was carried out with four hospitality graduate students and two foodservice experts. The questionnaire was slightly modified reflecting their feedback. Finally, the questionnaire was confirmed through the reviews by two professors, majoring foodservice.

### 3.2. Data collection

For data collection for this study, an online panel survey was conducted *via* the most prominent online panel survey firm in Korea. The samples were randomly chosen from the panel database of the firm through its random sampling method. Then, an email invitation was sent to the sampled panel members through the firm’s system. Through the link included in the invitation, the sampled members could access and fill out the online questionnaire. Only those who had dined in a restaurant within the last 5 months were eligible to complete the survey. If the participant decides to join this research, they were requested to sign an informed consent at the beginning of the questionnaire. This process produced 351 usable responses. Since there were no incomplete or inconsistent responses, all 351 responses were used for the analysis.

### 3.3. Sample profile

Among the 351 respondents, 51.0% were male and 49.0% were female customers. Their average age was 43.74, ranging from 20 to 69. In terms of age group, 23.4% were in the 40’s, 19.9.7% were in the 50’s, 19.4% were in the 20’s, 19.1% were in the 30’s, and 18.2% were in 60’s. Regarding the respondents’ education level, 65.0% had a bachelor’s degree, 14.5% reported graduate school or higher, 10.8% had a high school diploma, and 9.7% had an associate degree. About individual monthly income, 35.0% had an income lower than US$2,500, followed by between US$2,501 and 4,500 (31.6%), US$4,501–6,500 (18.3%), US$6,501–8,500 (8.3%), and over US$8,500 (6.8%). Overall, the samples appeared to well represent restaurant customers in Korea.

## 4. Results

### 4.1. Measurement model evaluation through confirmatory factor analysis

A CFA was performed to test the adequacy of the measurement model and data quality. As shown in Table 1, the model showed an adequate fit to the data (*χ*^2^ = 548.401 (*df* = 194, *p* < 0.001, *χ*^2^/*df* = 2.827), RMSEA = 0.072, CFI = 0.947, IFI = 0.947, TLI = 0.937). All the measures loaded on their relevant constructs at *p* < 0.001. The composite reliability of the constructs ranged from 0.841 to 0.936, which are all above the recommended minimum of 0.700 (Hair et al., 2010). The values of average variance extracted (AVE) ranged from 0.573 to 0.823, surpassing the required minimum of 0.500 (Hair et al., 2010). Accordingly, convergent validity of the measures was confirmed. Lastly, the AVE of each construct showed a higher value than all the squared correlations with the other constructs, showing adequate discriminant validity of the constructs (Hair et al., 2010).

**Table 1:** Results of the confirmatory factor analysis and correlations.

Construct	(a)	(b)	(c)	(d)	(e)	CR	AVE
(a) Nature connection	–	0.416^b^	0.582	0.240	0.277	0.936	0.648
(b) Biospheric values	0.645^a^	-	0.306	0.201	0.462	0.928	0.762
(c) Environmental self-identity	0.763	0.553	–	0.283	0.231	0.904	0.759
(d) Sense of obligation to reduce food leftover	0.490	0.448	0.532	–	0.501	0.841	0.573
(e) Food leftover reduction intention	0.526	0.680	0.481	0.708	–	0.933	0.823
Mean	5.06	6.05	5.05	4.83	5.72		
SD	1.04	0.94	1.03	1.19	1.09		
Goodness-of-fit statistics: *χ*^2^ = 548.401 (*df* = 194, *p* < 0.001, *χ*^2^/*df* = 2.827), RMSEA = 0.072, CFI = 0.947, IFI = 0.947, TLI = 0.937							

CR, composite reliability; AVE, average variance extracted; SD, standard deviation; RMSEA, root mean square error of approximation; CFI, comparative fit index; IFI, incremental fit index; TLI, Tucker-Lewis index. ^a^Correlation; ^b^Squared correlation.

### 4.2. Hypotheses testing through structural model analysis

**Figure 2:**
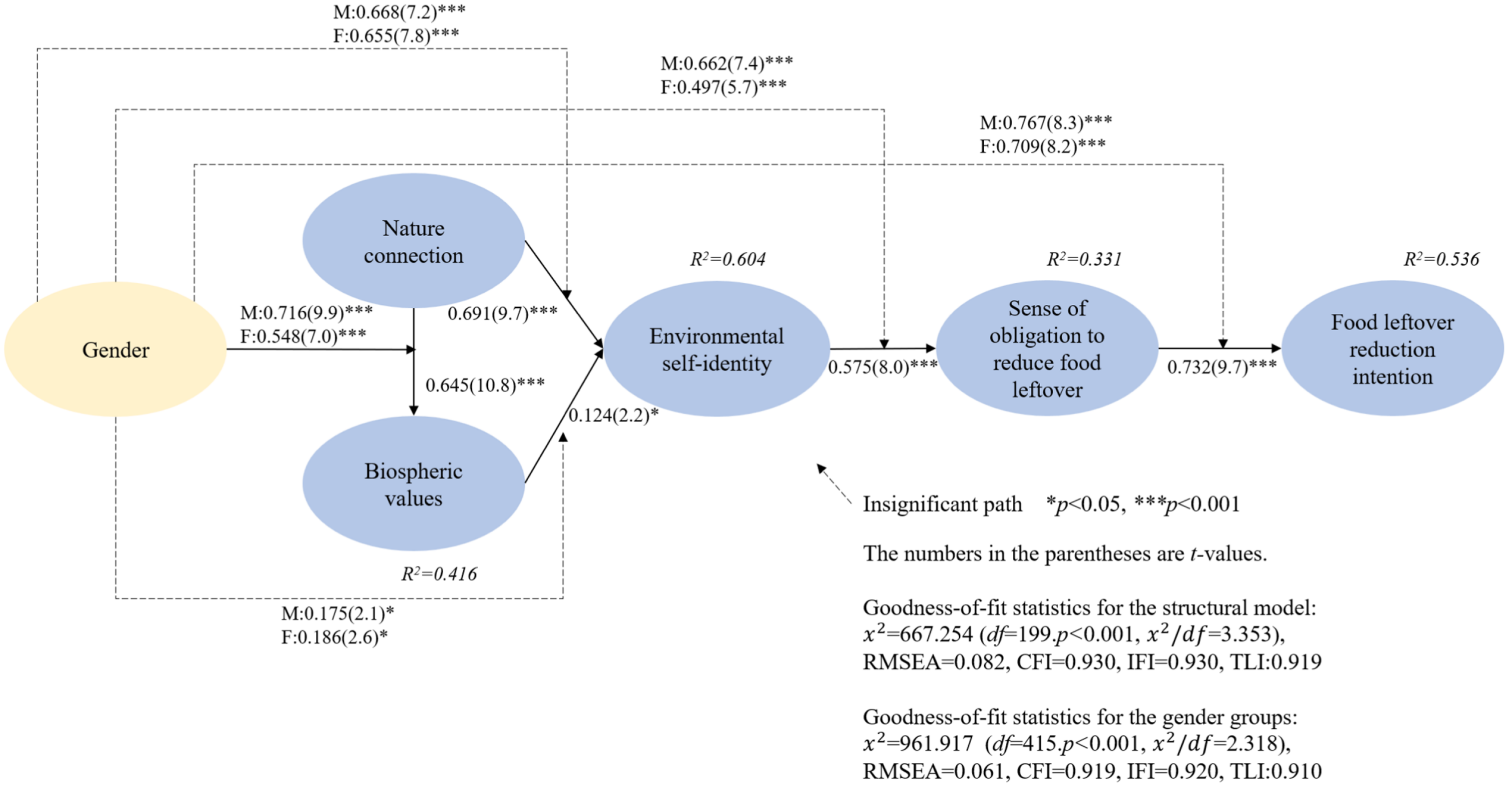
Results of structural model evaluation and baseline model estimation.

A structural equation modeling (SEM) was conducted with a maximum likelihood estimation method to assess the conceptual research model as shown in Table 2 and Figure 2. The structural model adequately fit the data (*χ*^2^ = 667.254 (*df* = 199, *p* < 0.001, *χ*^2^/*df* = 3.353), RMSEA = 0.082, CFI = 0.930, IFI = 0.930, TLI = 0.919). The model showed satisfactory variance explanation power for food leftover reduction intention (*R*^2^ = 53.6%).

**Table 2:** Results of the structural equation modeling (*n* = 351).

	Independent construct		Dependent construct	Coefficient	*t*-value
H1	Nature connection	**→**	Biospheric values	0.645	10.823^***^
H2	Nature connection	**→**	Environmental self-identity	0.691	9.699^***^
H3	Biospheric values	**→**	Environmental self-identity	0.124	2.206^*^
H4	Environmental self-identity	**→**	Sense of obligation to reduce food leftover	0.575	8.041^***^
H5	Sense of obligation to reduce food leftover	**→**	Food leftover reduction intention	0.732	9.655^***^
Total variance explained (*R*^2^): *R^2^* for biospheric values = 0.416. *R^2^* for environmental self-identity = 0.604. *R^2^* for sense of obligation to reduce food leftover = 0.331. *R^2^* for food leftover reduction intention = 0.536			Goodness-of-fit statistics: *χ*^2^ = 667.254 (*df* = 199, *p* < 0.001, *χ*^2^/*df* = 3.353), RMSEA = 0.082, CFI = 0.930, IFI = 0.930, TLI = 0.919		

RMSEA, root mean square error of approximation; CFI, comparative fit index; IFI, incremental fit index; TLI, Tucker-Lewis index.

^*^p < 0.05 and ^***^p < 0.001.

Regarding the hypothesized relationships, reported in Table 2 and Figure 2, the SEM outcomes showed that nature connection significantly positively affected biospheric values (*β* = 0.645, *p* < 0.001), supporting H1. Nature connection also significantly and positively affected environmental self-identity (*β* = 0.691, *p* < 0.001), supporting H2. Biospheric values also show a significant positive influence on environmental self-identity (*β* = 0.124, *p* < 0.05), supporting H3. In turn, environmental self-identity significantly and positively affected sense of obligation to reduce food leftover (*β* = 0.575, *p* < 0.001) and, lastly, sense of obligation to reduce food leftover significantly positively affected food leftover reduction intention (*β* = 0.732, *p* < 0.001), supporting H4 and H5.

As the next phase, the indirect effects of the variables were evaluated. This research used AMOS software to test the indirect effect (Byrne, 2010; Hair et al., 2010). As shown in Table 3, environmental self-identity indirectly affected food leftover reduction intention (*β* = 0.421, *p* < 0.01). Biospheric values indirectly influenced sense of obligation to reduce food leftover (*β* = 0.071, *p* < 0.01) and food leftover reduction intention (*β* = 0.052, *p* < 0.01). Nature connection indirectly affected environmental self-identity (*β* = 0.080, *p* < 0.01), sense of obligation to reduce food leftover (*β* = 0.444, *p* < 0.05), and food leftover reduction intention (*β* = 0.325, *p* < 0.01). Overall, the results showed that all the mediating variables in the structural model played significant roles. Lastly, regarding the total effects on food leftover reduction intention, the findings disclosed that sense of obligation to reduce food leftover had the strongest effect (*β* = 0.732, *p* < 0.01), followed by environmental self-identity (*β* = 0.421, *p* < 0.01), nature connection (*β* = 0.325, *p* < 0.05), and biospheric values (*β* = 0.052, *p* < 0.01).

**Table 3:** Results of the indirect and total effect assessment.

On	Indirect effect of		
	Nature connection	Biospheric values	Environmental self-identity
Environmental self-identity	0.080^**^	–	–
Sense of obligation to reduce food leftover	0.444^*^	0.071^**^	–
Food leftover reduction intention	0.325^**^	0.052^**^	0.421^**^
Total effect on food leftover reduction intention: *β* Nature connection = 0.325^*^ β Biospheric values = 0.052^**^ β Environmental self-identity = 0.421^**^ β Sense of obligation to reduce food leftover = 0.732^**^			

^*^p < 0.05 and ^**^p < 0.01.

### 4.3. Moderating effect by gender

A metric invariance test was carried out to test the hypothesized moderating effects of gender. First, all the respondents were separated into male group (*n* = 179) and female group (*n* = 172). In turn, a baseline model including all both male and female groups were examined. The test results disclosed that the baseline model fits the data well (*χ*^2^ = 961.917 (*df* = 415, *p* < 0.001, *χ*^2^/*df* = 2.318), RMSEA = 0.061, CFI = 0.919, IFI = 0.920, TLI = 0.910) as shown in Table 4 and Figure 2. Then, the baseline model was compared to a series of models in which a specific relationship was set to be equal. This process was to reveal how differently male and female customers’ pro-environmental attitude affects its dependent attitude variable. The invariance test revealed that the effects of nature connection on biospheric values were significantly different across the two gender groups, supporting H6a. However, unlike H6b-e, the other relationships were not significantly different across the gender groups.

**Table 4:** Results of the invariance model evaluation.

Linkage	Male group (*n* = 179)		Female group (*n* = 172)		Baseline model (freely estimated)	Nested model (equally constrained)
	*β*	*t*-value	*β*	*t*-value		
Nature connection → Biospheric values	0.716	9.889^***^	0.548	7.043^***^	*χ^2^*(415) = 961.917	*χ^2^*(416) = 972.219^a^
Nature connection → Environmental self-identity	0.668	7.203^***^	0.655	7.814^***^	*χ^2^*(415) = 961.917	*χ^2^*(416) = 961.921^b^
Biospheric values → Environmental self-identity	0.175	2.078^*^	0.186	2.570^**^	*χ^2^*(415) = 961.917	*χ^2^*(416) = 962.157^c^
Environmental self-identity → Sense of obligation to reduce food leftover	0.662	7.358^***^	0.497	5.734^***^	*χ^2^*(415) = 961.917	*χ^2^*(416) = 962.505^d^
Sense of obligation to reduce food leftover → Food leftover reduction intention	0.767	8.294^***^	0.709	8.219^***^	*χ^2^*(415) = 961.917	*χ^2^*(416) = 963.015^e^
Goodness-of-fit statistics for the baseline model for gender groups: *χ*^2^ = 961.917 (*df* = 415, *p* < 0.001, *χ*^2^/*df* = 2.318), RMSEA = 0.061, CFI = 0.919, IFI = 0.920, TLI = 0.910						

*χ*^2^ difference test: ^a^*Δχ*^2^(1) = 10.302, *p* < 0.01 (H1: Supported); ^b^*Δχ*^2^(1) = 0.004, *p* > 0.05 (H2: Not supported); ^c^*Δχ*^2^(1) = 0.240, *p* > 0.05 (H3: Not supported); ^d^*Δχ*^2^(1) = 0.588, *p* > 0.05 (H4: Not supported); ^e^*Δχ*^2^(1) = 1.098, *p* > 0.05 (H5: Not supported). ^*^p < 0.05, ^**^p < 0.01, ^***^p < 0.001.

## 5. Discussion

### 5.1. Theoretical implications

The statistical results revealed that all the hypothesized direct effects were significant in this research. To our best knowledge, this study is the first empirical attempt to explain how restaurant customers’ food leftover reduction intention is formed through the human-nature relationship, which was represented by nature connection, biospheric values, and environmental self-identity. Given that food waste not only harms the natural environment (e.g., Rico et al., 2019) but also diminishes restaurants’ profitability, the findings of this study provide valuable insights to policymakers and food service practitioners. Specifically, in the restaurant context, nature connection was observed to be a significant predictor of biospheric values and environmental identity, explaining a fairly large portion of the variance of biospheric values. Further, with biospheric values, nature connection accounted for a large portion of the variance of environmental identity. These results imply that if a customer feels connected or oneness with nature, he/she is more likely to judge issues based on costs or benefits to the biosphere. In addition, such a customer is more likely to define him−/herself as an eco-friendly person or as an environmentalist. As such, these findings indicate that nature connection is a crucial variable in fostering restaurant customers’ environmental self-identity, leading to their food leftover reduction intention. The findings of this study revealed that restaurant customers’ environmental self-identity significantly strengthened their sense of obligation to reduce food leftover with a relatively high prediction power. In turn, their sense of obligation to reduce food leftover significantly heightened their food leftover reduction intention, determining a large amount of the variance of their food leftover reduction intention.

### 5.2. Practical implication

Previous research has noted that biospheric values are essential to citizens’ environment-friendly attitudes and behaviors (Martin and Czellar, 2017). Furthermore, several studies report the relationship between a sense of obligation and food leftover reduction intention (Fielding et al., 2008; Schutte and Malouff, 2018; Stancu and Lähteenmäki, 2022). the current study revealed the specific roles of nature connection, biospheric values, and environmental self-identity in fostering restaurant customers’ sense of obligation to reduce food leftover and food leftover reduction intention. Therefore, food service providers can reduce customer food leftover, which takes up 75% of food waste in food services in the case of the EU (Oliveira et al., 2016), by arousing customers’ nature connection and biospheric values. Jagau and Vyrastekova (2017) observed that customers seemed to feel shame *via* food waste reduction posters when they left food on their plates. Pinto et al. (2018) displayed a food waste reduction poster in the canteen and observed that students left less food than usual. For food services, reducing food leftover will not only directly save costs on food materials and food waste disposal but also indirectly contribute to their profitability through increased customer traffic owing to the improved image as environment-friendly restaurants (Han, 2020). Such an image is decisive in this era of an eco-friendly economy (Han, 2020). Regarding food waste in food services, the impact of customers’ behavioral change is substantial compared to the efforts required, such as ordering just the right amount of food and taking leftovers home. Arousing customers’ oneness feeling with nature and appealing to their biospheric values can be used to engage customers in food leftover prevention actively. Thus, this approach is respecting their conscious choice to reduce food leftover, coming from their goodwill toward nature.

In addition, this study suggests that enhancing citizens’ nature connection can effectively cultivate biospheric values in their minds. Steg and de Groot (2012) argued that biospheric values in citizens should be cultivated to promote their pro-environmental behaviors. Miller (2005) suggested that less time in nature means less opportunity to develop a nature connection, which leads to a less caring attitude and behavior toward nature. On the flip side, if people spend more time in nature early on, they can develop a closer relationship with nature (Rickard and White, 2021). Unfortunately, more and more children are losing opportunities to experience nature due to increasing urbanization and addictive online activities. Activities and relationships in the online world are fascinating to children. However, nature is also inherently appealing to children. They innately love nature and know how to play in nature with its components like animals, trees, pebbles, sand, and water (Kellert, 2002). Activities in nature benefits children emotionally and physically, which is called well-being (Kahn and Kellert, 2002). Through activities in nature, children can make good memories and develop a relationship with nature (Hughes et al., 2018), which, in turn, will lead them to care for nature throughout their lifetime, as previous research and this study indicate. Indeed, this human-nature relationship applies to all humans regardless of age, as humans were born and evolved in nature (Wilson, 1984). Therefore, from a long-term point of view, policymakers may develop supporting programs to give children more chances to interact and develop a relationship with nature to enhance their natural connection. As mentioned, it is also emotionally and physically beneficial to children, especially in metropolitan areas, where children are relatively away from nature. In that way, children can learn how to live together in connection with nature. In environmental psychology, this research contributes to the management and development of consumers’ pro-environmental behaviors, such as improving the sense of obligation to reduce food leftovers.

### 5.3. Limitations and future research

This study has a few limitations. First, the samples of this study included only Korean customers using Korean restaurants. For this reason, the generalizability of the findings in this study is limited. Thus, future research may extend this study by using a more comprehensive range of samples from various countries. Second, the data used in this study were collected during the COVID-19 pandemic. This pandemic might lead people to have higher biospheric values only, for the time being, owing to the belief that the pandemic was caused by environmental destruction (Chua et al., 2020). In this regard, future research should extend this study by asking for customers’ responses during the post-Corona period. Third, this study is a cross-sectional study. Thus, readers should interpret the findings of this study with caution by keeping the nature of this study in mind. Future research may test a similar research model with a longitudinal approach to supplement our findings. Lastly, for generalizability, the influence of restaurant types was not considered in this study. If future researchers are interested in type-specific results or differences in results across different types, they may include restaurant types as a predictor variable.

### 5.4. Conclusion

This study is set to explore the promotive effects of nature connection and biospheric values in motivating restaurant customers’ food leftover reduction intention through environmental self-identity and a sense of obligation to reduce food leftover. The result of this study suggests that enhancing citizens’ nature connection can effectively cultivate biospheric values in their minds. Overall, the current research strengthens the idea that individuals connected to nature are more likely to form food leftover reduction intention, owing to a caring mind toward nature. This research also found that gender moderates the impact of nature connection on biospheric values. Specifically, nature connection more strongly affected biospheric values for men than women. This result implies that when perceiving a similar level of nature connection, men generate more vital biospheric values than women. Given that this study is the first to test the gender difference in this relationship, this finding broadened our understanding of the gender role in the pro-environment domain. From the practical perspective, this finding may be used in designing campaign messages that stimulate restaurant customers’ biospheric values targeting more men than women because that would be more effective than the opposite.

## Data availability statement

The raw data supporting the conclusions of this article will be made available by the authors, without undue reservation.

## Ethics statement

Ethical review and approval was not required for the study on human participants in accordance with the local legislation and institutional requirements. Written informed consent from the patients/ participants or patients/participants legal guardian/next of kin was not required to participate in this study in accordance with the national legislation and the institutional requirements.

## Author contributions

All authors listed have made a substantial, direct, and intellectual contribution to the work and approved it for publication.

## Conflict of interest

The authors declare that the research was conducted in the absence of any commercial or financial relationships that could be construed as a potential conflict of interest.

## Publisher’s note

All claims expressed in this article are solely those of the authors and do not necessarily represent those of their affiliated organizations, or those of the publisher, the editors and the reviewers. Any product that may be evaluated in this article, or claim that may be made by its manufacturer, is not guaranteed or endorsed by the publisher.

## Appendix 1. Measurement items

**Table:**

Constructs	Items	Standardized loading
Nature Connection	I often feel a sense of oneness with the natural world around me.	0.726
	I think of the natural world as a community to which I belong.	0.816
	I recognize and appreciate the intelligence of other living organisms.	0.750
	When I think of my life, I imagine myself to be part of a larger cyclical process of living.	0.840
	I have a deep understanding of how my actions affect the natural world.	0.797
	I feel that all inhabitants of Earth, human, and nonhuman, share a common ‘life force’.	0.843
	Like a tree can be part of a forest, I feel embedded within the broader natural world.	0.865
	My personal welfare is dependent of the welfare of the natural world.	0.795
Bioshperic Values	We human beings should not damage the beauty of the nature.	0.872
	We human beings should live harmoniously with the nature.	0.896
	The nature is very beautiful.	0.858
	It would be a shame if we human beings damage the environment for our benefits.	0.866
Environmental Self-identity	Acting environmentally-friendly is an important part of who I am.	0.794
	I am the type of person who acts environmentally-friendly.	0.910
	I see myself as an environmentally-friendly person.	0.905
Sense of Obligation to Reduce Food Leftover	I feel guilty about poor people when I leave leftover food.	0.614
	Leaving leftovers give me a bad conscience.	0.769
	I have been raised to eat all food I have taken myself.	0.764
	It is contrary my principles when I have to discard food.	0.860
Food Leftover Reduction Intention	I try to eat all food that I have ordered.	0.921
	I intend to not throw food away.	0.923
	I try to leave as little food as possible.	0.877

All the loadings were significant at *p* < 0.001.

## References

[ref1] Abdelradi F. (2018). Food waste behaviour at the household level: a conceptual framework. Waste Manag. 71, 485–493. doi: 10.1016/j.wasman.2017.10.001, PMID: 29037881

[ref2] Adelodun B. Kim S. H. Odey G. Choi K. S. (2021). Assessment of environmental and economic aspects of household food waste using a new environmental-economic footprint (EN-EC) index: a case study of Daegu, South Korea. Sci. Total. Environ. 776:145928. doi: 10.1016/j.scitotenv.2021.145928, PMID: 33640543

[ref3] Ajzen I. (1991). The theory of planned behaviour. Organ. Behav. Hum. Decis. Process. 50, 179–211. doi: 10.1016/0749-5978(91)90020-T

[ref4] Amicarelli V. Bux C. (2020). Food waste measurement toward a fair, healthy and environmental-friendly food system: a critical review. Br. Food. J. 123, 2907–2935. doi: 10.1108/BFJ-07-2020-0658

[ref5] Amicarelli V. Lagioia G. Sampietro S. Bux C. (2021). Has the COVID-19 pandemic changed food waste perception and behavior? Evidence from Italian consumers. Socio-Econ. Plan. Sci. 101095. 10.1016/j.seps.2021.101095 PMC9751389 36536871

[ref6] Ananda J. Karunasena G. G. Mitsis A. Kansal M. Pearson D. (2021). Analysing behavioural and socio-demographic factors and practices influencing Australian household food waste. J. Clean. Prod. 306:127280. doi: 10.1016/j.jclepro.2021.127280

[ref7] Attiq S. Habib M. D. Kaur P. Hasni M. J. S. Dhir A. (2021). Drivers of food waste reduction behaviour in the household context. Food Qual. Prefer. 94:104300. doi: 10.1016/j.foodqual.2021.104300

[ref8] Baldwin J. D. (2002). George Herbert Mead: A Unifying Theory for Sociology. New York, NY: Kendall.

[ref9] Barr S. Gilg A. W. Ford N. J. (2001). A conceptual framework for understanding and analyzing attitudes towards household-waste management. Environ. Plan. A 33, 2025–2048. doi: 10.1068/a33225

[ref10] Birindelli G. Iannuzzi A. P. Savioli M. (2019). The impact of women leaders on environmental performance: evidence on gender diversity in banks. Corp. Soc. Responsib. Environ. Manag. 26, 1485–1499. doi: 10.1002/csr.1762

[ref11] Briscoe M. D. Givens J. E. Hazboun S. O. Krannich R. S. (2019). At home, in public, and in between: gender differences in public, private and transportation pro-environmental behaviors in the US intermountain west. Environ. Sociol. 5, 374–392. doi: 10.1080/23251042.2019.1628333

[ref12] Bux C. Amicarelli V. (2022). Material flow cost accounting (MFCA) to enhance environmental entrepreneurship in the meat sector: challenges and opportunities. J. Environ. Manag. 313:115001. doi: 10.1016/j.jenvman.2022.115001, PMID: 35381529

[ref13] Buzby J. C. Farah-Wells H. Hyman J. (2014). The estimated amount, value, and calories of postharvest food losses at the retail and consumer levels in the United States. USDA-ERS economic information. Bulletin 121, 1–33. doi: 10.2139/ssrn.2501659

[ref14] Byrne B. M. (2010). Structural Equation Modeling With AMOS: Basic Concepts, Applications, and Programming (2nd *edn.*). Mahwah, NJ: Erlbaum.

[ref15] Capaldi C. A. Dopko R. L. Zelenski J. M. (2014). The relationship between nature connectedness and happiness: a meta-analysis. Front. Psychol. 5:976. doi: 10.3389/fpsyg.2014.00976, PMID: 25249992 PMC4157607

[ref16] Chen C. C. Sujanto R. Y. Tseng M. L. Fujii M. Lim M. K. (2021). Sustainable consumption transition model: social concerns and waste minimization under willingness-to-pay in Indonesian food industry. Resour. Conserv. Recycl. 170:105590. doi: 10.1016/j.resconrec.2021.105590

[ref17] Christ K. L. Burritt R. (2017). Material flow cost accounting for food waste in the restaurant industry. Br. Food J. 119, 600–612. doi: 10.1108/BFJ-07-2016-0318

[ref18] Chua B. L. Al-Ansi A. Lee M. J. Han H. (2020). Impact of health risk perception on avoidance of international travel in the wake of a pandemic. Curr. Issues Tour. 24, 985–1002.

[ref19] Coşkun A. Özbük R. M. Y. (2020). What influences consumer food waste behavior in restaurants? An application of the extended theory of planned behavior. Waste Manag. 117, 170–178. doi: 10.1016/j.wasman.2020.08.011, PMID: 32861079

[ref20] Earth. (2021). How South Korea became an example of how to recycle food waste. Available at: https://earth.org/food-waste-south-korea/ (Accessed June 4, 2022).

[ref21] Elhoushy S. (2020). Consumers’ sustainable food choices: antecedents and motivational imbalance. Int. J. Hosp. Manag. 89:102554. doi: 10.1016/j.ijhm.2020.102554

[ref22] Ertz M. Favier R. Robinot É. Sun S. (2021). To waste or not to waste? Empirical study of waste minimization behavior. Waste Manag. 131, 443–452. doi: 10.1016/j.wasman.2021.06.032 34256344

[ref23] Evans D. (2012). Binning, gifting and recovery: the conduits of disposal in household food consumption. Environ. Plan. D: Soc. Space. 30, 1123–1137. doi: 10.1068/d22210

[ref24] Fekadu Z. Kraft P. (2001). Self-identity in planned behavior perspective: past behavior and its moderating effects on self-identity-intention relations. Soc. Behav. Personal. 29, 671–685. doi: 10.2224/sbp.2001.29.7.671

[ref25] Fielding K. S. McDonald R. Louis W. R. (2008). Theory of planned behaviour, identity and intentions to engage in environmental activism. J. Environ. Psychol. 28, 318–326. doi: 10.1016/j.jenvp.2008.03.003

[ref26] Filimonau V. Fidan H. Alexieva I. Dragoev S. Marinova D. D. (2019). Restaurant food waste and the determinants of its effective management in Bulgaria: an exploratory case study of restaurants in Plovdiv. Tour. Manag. Perspect. 32:100577. doi: 10.1016/j.tmp.2019.100577

[ref27] Food and Agriculture Organization of the United Nations (2021). South Korea has almost zero food waste, Here’s how. Available at: https://www.ahgingos.org/south-korea-has-almost-zero-food-waste-heres-how/ (Accessed June 4, 2022).

[ref28] Food and Agriculture Organization of the United Nations (2022). Sustainable development goals. Available at: https://www.fao.org/sustainable-development-goals/indicators/1231/en/ (Accessed June 4, 2022).

[ref29] Garrone P. Melacini M. Perego A. (2014). Opening the black box of food waste reduction. Food Policy 46, 129–139. doi: 10.1016/j.foodpol.2014.03.014

[ref30] Giordano C. Alboni F. Falasconi L. (2019). Quantities, determinants, and awareness of households’ food waste in Italy: a comparison between diary and questionnaires quantities. Sustainability. 11:3381. doi: 10.3390/su11123381

[ref31] Giordano C. Piras S. Boschini M. Falasconi L. (2018). Are questionnaires a reliable method to measure food waste? A pilot study on Italian households. Br. Food J. 2018, 2885–2897.

[ref32] Godfray H. C. J. Crute I. R. Haddad L. Lawrence D. Muir J. F. Nisbett N.. (2010). The future of the global food system. Philos. Trans. R Soc. Lond. B Biol. Sci. 365, 2769–2777. doi: 10.1098/rstb.2010.0180, PMID: 20713383 PMC2935131

[ref33] Gössling S. (2015). New performance indicators for water management in tourism. Tour. Manag. 46, 233–244. doi: 10.1016/j.tourman.2014.06.018

[ref34] Graham-Rowe E. Jessop D. C. Sparks P. (2014). Identifying motivations and barriers to minimising household food waste. Resour. Conserv. Recycl. 84, 15–23. doi: 10.1016/j.resconrec.2013.12.005

[ref35] Graham-Rowe E. Jessop D. C. Sparks P. (2015). Predicting household food waste reduction using an extended theory of planned behaviour. Resour. Conserv. Recycl. 101, 194–202. doi: 10.1016/j.resconrec.2015.05.020

[ref36] Hair J. F. Black W. C. Babin B. J. Anderson R. E. (2010). Multivariate Data Analysis, 7th *Edn*. Hoboken, NJ: Prentice-Hall.

[ref37] Hair J. F. Hult G. T. M. Ringle C. M. Sarstedt M. (2014). A Primer on Partial Least Squares Structural Equation Modeling (PLS-SEM). Thousand Oaks, CA: Sage Publications.

[ref38] Han H. (2020). Theory of green purchase behavior (TGPB): a new theory for green hotel and green restaurant products. Bus. Strat. Environ. 29, 2815–2828. doi: 10.1002/bse.2545

[ref39] Hong I. Park S. Lee B. Lee J. Jeong D. Park S. (2014). IoT-based smart garbage system for efficient food waste management. Sci. World J. 2014:646953. doi: 10.1155/2014/646953, PMID: 25258730 PMC4166430

[ref40] Howell A. J. Passmore H. A. Buro K. (2013). Meaning in nature: meaning in life as a mediator of the relationship between nature connectedness and well-being. J. Happiness Stud. 14, 1681–1696. doi: 10.1007/s10902-012-9403-x

[ref41] Hughes J. Richardson M. Lumber R. (2018). Evaluating connection to nature and the relationship with conservation behaviour in children. J. Nat. Conserv. 45, 11–19. doi: 10.1016/j.jnc.2018.07.004

[ref42] Jagau H. L. Vyrastekova J. (2017). Behavioral approach to food waste: an experiment. Br. Food J. 119, 882–894. doi: 10.1108/BFJ-05-2016-0213

[ref43] Jans L. (2021). Changing environmental behaviour from the bottom up: the formation of pro-environmental social identities. J. Environ. Psychol. 73:101531. doi: 10.1016/j.jenvp.2020.101531

[ref44] Kahn P. H. Kellert S. R. (eds) (2002). Children and Nature: Psychological, Sociocultural, and Evolutionary Investigations. Cambridge, MA: MIT Press.

[ref45] Kamau B. W. (2020). The intervening role of Employee's awareness on the relationship between the adequacy of welfare and job stability of university catering employees in Nairobi City county Kenya. J. Hosp. Tour. Manag. 3, 1–24.

[ref46] Katsarova I. (2014). Tackling Food Waste: The EU’s Contribution to a Global Issue; European Parliamentary Research Service (EPRS). France: Strasbourg.

[ref47] Kellert S. R. (2002). “Experiencing nature: Affective, cognitive, and evaluative development in children,” in Children and Nature: Psychological, Sociocultural, and Evolutionary Investigations. eds. Kahn P. H. Jr. Kellert S. R. (Cambridge, MA: MIT Press), 117–151.

[ref48] Kim S. H. Kim M. S. Lee M. S. Park Y. S. Lee H. J. Kang S. A.. (2016). Korean diet: characteristics and historical background. J. Ethn. Foods. 3, 26–31. doi: 10.1016/j.jef.2016.03.002

[ref49] Kwon D. Y. Chung K. R. Yang H. J. Jang D. J. (2015). Gochujang (Korean red pepper paste): a Korean ethnic sauce, its role and history. J. Ethn. Foods 2, 29–35. doi: 10.1016/j.jef.2015.02.006

[ref50] KZWMN. (2022). Introduction. Available at: http://www.waste21.or.kr (Accessed June 4, 2022).

[ref51] Lacasse K. (2016). Don't be satisfied, identify! Strengthening positive spillover by connecting pro-environmental behaviors to an “environmentalist” label. J. Environ. Psychol. 48, 149–158. doi: 10.1016/j.jenvp.2016.09.006

[ref52] Mannetti L. Pierro A. Livi S. (2004). Recycling: planned and self-expressive behaviour. J. Environ. Psychol. 24, 227–236. doi: 10.1016/j.jenvp.2004.01.002

[ref53] Martin C. Czellar S. (2017). Where do biospheric values come from? A connectedness to nature perspective. J. Environ. Psychol. 52, 56–68. doi: 10.1016/j.jenvp.2017.04.009

[ref54] Martin-Rios C. Demen-Meier C. Gössling S. Cornuz C. (2018). Food waste management innovations in the foodservice industry. Waste Manag. 79, 196–206. doi: 10.1016/j.wasman.2018.07.033, PMID: 30343746

[ref55] Mayer F. S. Frantz C. M. (2004). The connectedness to nature scale: a measure of individuals’ feeling in community with nature. J. Environ. Psychol. 24, 503–515. doi: 10.1016/j.jenvp.2004.10.001

[ref56] Miller J. R. (2005). Biodiversity conservation and the extinction of experience. Trend. Ecol. Evolut. 20, 430–434. doi: 10.1016/j.tree.2005.05.013 16701413

[ref57] Moraes N. V. Lermen F. H. Echeveste M. E. S. (2021). A systematic literature review on food waste/loss prevention and minimization methods. J. Environ. Manag. 286:112268. doi: 10.1016/j.jenvman.2021.112268, PMID: 33684802

[ref58] Moreton S. G. Arena A. Hornsey M. J. Crimston C. R. Tiliopoulos N. (2019). Elevating nature: moral elevation increases feelings of connectedness to nature. J. Environ. Psychol. 65:101332. doi: 10.1016/j.jenvp.2019.101332

[ref59] Nisbet E. K. Zelenski J. M. Murphy S. A. (2009). The nature relatedness scale: linking individuals' connection with nature to environmental concern and behavior. Environ. Behav. 41, 715–740. doi: 10.1177/0013916508318748

[ref60] Oh R. R. Y. Fielding K. S. Nghiem L. T. P. Chang C. C. Carrasco L. R. Fuller R. A. (2021). Connection to nature is predicted by family values, social norms and personal experiences of nature. Glob. Ecol. Conserv. 28:e01632. doi: 10.1016/j.gecco.2021.e01632

[ref61] Oliveira B. de Moura A. P. Cuhna L. M. (2016). “Reducing food waste in the food service sector as a way to promote public health and environmental sustainability,” in Climate Change and Health Book Subtitle: Improving Resilience and Reducing Risks. eds. Filho W. L. Azeiteiro U. M. Alves F. (Germany: Springer), 117–132.

[ref62] Öztayşi B. Baysan S. Akpinar F. (2009). Radio frequency identification (RFID) in hospitality. Technovation 29, 618–624. doi: 10.1016/j.technovation.2009.05.014

[ref63] Pinto R. S. dos Santos Pinto R. M. Melo F. F. S. Campos S. S. Cordovil C. M. D. S. (2018). A simple awareness campaign to promote food waste reduction in a university canteen. Waste Manag. 76, 28–38. doi: 10.1016/j.wasman.2018.02.044, PMID: 29503053

[ref64] Principato L. Di Leo A. Mattia G. Pratesi C. A. (2021). The next step in sustainable dining: the restaurant food waste map for the management of food waste. Ital. J. Mark. 2021, 189–207. doi: 10.1007/s43039-021-00032-x

[ref65] Quested T. Ingle R. Parry A. (2013). Household Food and Drink Waste in the United Kingdom 2012. United Kingdom: WRAP.

[ref66] Rand D. G. Brescoll V. L. Everett J. A. Capraro V. Barcelo H. (2016). Social heuristics and social roles: intuition favors altruism for women but not for men. J. Exp. Psychol. Gen. 145, 389–396. doi: 10.1037/xge0000154, PMID: 26913619

[ref67] Rickard S. C. White M. P. (2021). Barefoot walking, nature connectedness and psychological restoration: the importance of stimulating the sense of touch for feeling closer to the natural world. Landsc. Res. 46, 975–991. doi: 10.1080/01426397.2021.1928034

[ref68] Rico A. Martínez-Blanco J. Montlleó M. Rodríguez G. Tavares N. Arias A.. (2019). Carbon footprint of tourism in Barcelona. Tour. Manag. 70, 491–504. doi: 10.1016/j.tourman.2018.09.012

[ref69] Sakaguchi L. Pak N. Potts M. D. (2018). Tackling the issue of food waste in restaurants: options for measurement method, reduction and behavioral change. J. Clean. Prod. 180, 430–436. doi: 10.1016/j.jclepro.2017.12.136

[ref70] Schanes K. Dobernig K. Gözet B. (2018). Food waste matters-a systematic review of household food waste practices and their policy implications. J. Clean. Prod. 182, 978–991. doi: 10.1016/j.jclepro.2018.02.030

[ref71] Schutte N. S. Malouff J. M. (2018). Mindfulness and connectedness to nature: a meta-analytic investigation. Pers. Individ. Dif. 127, 10–14. doi: 10.1016/j.paid.2018.01.034

[ref72] Schwartz S. H. (1977). “Normative influences on altruism,” in Advances in Experimental Social Psychology. ed. Berkowitz L. (Cambridge, MA: Academic Press), 221–279.

[ref73] Secondi L. Principato L. Laureti T. (2015). Household food waste behaviour in EU-27 countries: a multilevel analysis. Food Policy 56, 25–40. doi: 10.1016/j.foodpol.2015.07.007

[ref74] Shimanoff S. B. (2009). Gender Role Theory. Encyclopedia of Communication Theory. Thousand Oaks, CA: SAGE Publications, Inc.

[ref75] Stancu V. Lähteenmäki L. (2022). Consumer-related antecedents of food provisioning behaviors that promote food waste. Food Policy 108:102236. doi: 10.1016/j.foodpol.2022.102236

[ref76] Steg L. de Groot J. I. (2012). “Environmental values” in The Oxford Handbook of Environmental and Conservation Psychology. ed. Clayton S. (New York, NY: Oxford University Press), 81–92.

[ref77] Steg L. Perlaviciute G. Van der Werff E. Lurvink J. (2014). The significance of hedonic values for environmentally relevant attitudes, preferences, and actions. Environ. Behav. 46, 163–192. doi: 10.1177/0013916512454730

[ref78] Stern P. C. Dietz T. (1994). The value basis of environmental concern. J. Soc. Issues 50, 65–84. doi: 10.1111/j.1540-4560.1994.tb02420.x

[ref79] Stern P. C. Dietz T. Abel T. D. Guagnano G. A. Kalof L. (1999). A value-belief-norm theory of support for social movements: the case of environmentalism. Hum. Ecol. Rev. 6, 81–97.

[ref80] Stöckli S. Dorn M. Liechti S. (2018). Normative prompts reduce consumer food waste in restaurants. Waste Manag. 77, 532–536. doi: 10.1016/j.wasman.2018.04.047, PMID: 29731405

[ref81] Stryker S. (1980). Symbolic Interactionism: A Social Structural Version. Menlo Park, CA: Benjamin Cummings.

[ref82] Thompson B. (2004). Exploratory and Confirmatory Factor Analysis: Understanding Concepts and Applications. Washington, DC: American psychological Association.

[ref83] Thyberg K. L. Tonjes D. J. (2016). Drivers of food waste and their implications for sustainable policy development. Resour. Conser. Recycl. 106, 110–123. doi: 10.1016/j.resconrec.2015.11.016

[ref84] Tonglet M. Phillips P. S. Read A. D. (2004). Using the theory of planned behaviour to investigate the determinants of recycling behaviour: a case study from Brixworth, UK. Resour. Conserv. Recycl. 41, 191–214. doi: 10.1016/j.resconrec.2003.11.001

[ref85] Trelohan M. (2022). Do women engage in pro-environmental behaviours in the public sphere due to social expectations? The effects of social norm-based persuasive messages. VOLUNTAS: Int. J. Volunt. Nonprofit. Organ. 33, 134–148. doi: 10.1007/s11266-020-00303-9

[ref86] Ullman J. B. Bentler P. M. (2012). Structural Equation Modeling. Handbook of Psychology, 2nd *Edn.* Hoboken, NJ: Wiley Online Library.

[ref87] Van der Werff E. Steg L. Keizer K. (2013a). It is a moral issue: the relationship between environmental self-identity, obligation-based intrinsic motivation and pro-environmental behaviour. Glob. Environ. Chang. 23, 1258–1265. doi: 10.1016/j.gloenvcha.2013.07.018

[ref88] Van der Werff E. Steg L. Keizer K. (2013b). The value of environmental self-identity: the relationship between biospheric values, environmental self-identity and environmental preferences, intentions and behaviour. J. Environ. Psychol. 34, 55–63. doi: 10.1016/j.jenvp.2012.12.006

[ref89] Visschers V. H. Wickli N. Siegrist M. (2016). Sorting out food waste behaviour: a survey on the motivators and barriers of self-reported amounts of food waste in households. J. Environ. Psychol. 45, 66–78. doi: 10.1016/j.jenvp.2015.11.007

[ref90] Von Kameke C. Fischer D. (2018). Preventing household food waste via nudging: an exploration of consumer perceptions. J. Clean. Prod. 184, 32–40. doi: 10.1016/j.jclepro.2018.02.131

[ref91] Wang L. Liu G. Liu X. Liu Y. Gao J. Zhou B.. (2017). The weight of unfinished plate: a survey based characterization of restaurant food waste in Chinese cities. Waste Manag. 66, 3–12. doi: 10.1016/j.wasman.2017.04.007, PMID: 28438432

[ref92] Wen Z. Hu S. De Clercq D. Beck M. B. Zhang H. Zhang H.. (2018). Design, implementation, and evaluation of an internet of things (IoT) network system for restaurant food waste management. Waste Manag. 73, 26–38. doi: 10.1016/j.wasman.2017.11.054, PMID: 29242117

[ref93] Whitmarsh L. O'Neill S. (2010). Green identity, green living? The role of pro-environmental self-identity in determining consistency across diverse pro-environmental behaviours. J. Environ. Psychol. 30, 305–314. doi: 10.1016/j.jenvp.2010.01.003

[ref94] Wilson E. O. (1984). Biophilia. Cambridge, MA: Harvard University Press.

[ref95] World Food Programme. (2022). WFP global update on COVID-19. Available at: https://www.wfp.org/emergencies/covid-19-pandemic (Accessed June 4, 2022).

